# Neither *MICA* Nor *DEPDC5* Genetic Polymorphisms Correlate with Hepatocellular Carcinoma Recurrence following Hepatectomy

**DOI:** 10.1155/2012/185496

**Published:** 2012-10-24

**Authors:** Takashi Motomura, Yuki Ono, Ken Shirabe, Takasuke Fukuhara, Hideyuki Konishi, Yohei Mano, Takeo Toshima, Shohei Yoshiya, Jun Muto, Toru Ikegami, Tomoharu Yoshizumi, Yoshihiko Maehara

**Affiliations:** Department of Surgery and Science, Graduate School of Medical Sciences, Kyushu University, Fukuoka 812-8582, Japan

## Abstract

*Purpose*. Genetic polymorphisms of *MICA* and *DEPDC5* have been reported to correlate with progression to hepatocellular carcinoma (HCC) in chronic hepatitis C patients. However, correlation of these genetic variants with HCC recurrence following hepatectomy has not yet been clarified. *Methods*. Ninety-six consecutive HCC patients who underwent hepatectomy, including 64 patients who were hepatitis C virus (HCV) positive, were genotyped for *MICA* (rs2596542) and *DEPDC5* (rs1012068). Recurrence-free survival rates (RFS) were compared for each genotype. *Results*. Five-year HCC recurrence-free survival (RFS) rates following hepatectomy were 20.7% in *MICA* GG allele carriers, 38.7% in GA, and 20.8% in AA, respectively (*P* = 0.72). The five-year RFS rate was 23.8% in *DEPDC5* TT allele carriers and 31.8% in TG/GG, respectively (*P* = 0.47). The survival rates in all (including HCV-negative) patients were also similar among each *MICA* and *DEPDC5* genotype following hepatectomy. Among HCV-positive patients carrying the *DEPDC5* TG/GG allele, low fibrosis stage (F0-2) occurred more often compared with TT carriers (*P* < 0.05). *Conclusions*. Neither *MICA* nor *DEPDC5* genetic polymorphism correlates with HCC recurrence following hepatectomy. *DEPDC5* minor genotype data suggest a high susceptibility for HCC development in livers, even those with low fibrosis stages.

## 1. Introduction

Hepatocellular carcinoma (HCC) is among the top five causes of cancer-related death worldwide [1] and is ranked third in the leading cause of death from cancer in Japanese men, of which approximately 70% is related with hepatitis C virus (HCV) infection [2]. Despite the recent development of cancer therapies, such as surgical resection, liver transplantation, local ablation, arterial embolization, and tyrosine kinase inhibitors, almost 70% of patients will relapse within 5 years after initial surgical resection [3]. In particular, a significant association between the histological hepatitis states of the remnant livers and HCC recurrence has been reported. High histological hepatitis activity and high serum levels of transaminase are related to a high recurrence rate, especially in HCV patients. This recurrence is presumed to be caused by multicentric occurrence of HCC after hepatectomy in patients with HCV [4, 5]. 

Recent technical developments in genome sequencing have enabled genome-wide association studies, which clarified that two single nucleotide polymorphisms (SNPs) are associated with progression to HCC among HCV patients. The SNPs are in the *MICA* [6] and *DEPDC5* genes [7]. The *MICA* gene encodes a major histocompatibility complex class I polypeptide-related sequence A, which is a membrane protein that activates the anticancer effect of natural killer cells or CD8 positive T cells [8]. In the previous report [6], A allele at rs2596542 (G/A) was shown to be more susceptible for development of HCC with 1.39 of odds ratio. On the other hand, the function of *DEPDC5* remains unknown, but is suggested to correlate with several other cancers [9]. G allele at rs1012068 (T/G) was reported to correlate with progression to HCC with 1.75 of odds ratio [7]. Taken together, it could be assumed that SNPs in *MICA* and *DEPDC5* may also correlate with HCC recurrence after curative therapies among HCV patients, or even among the other patients suffering from various kinds of liver diseases.

In this study, we investigated whether *MICA* and *DEPDC5* genetic polymorphisms were significant prognostic factors for HCC recurrence following hepatectomy. 

## 2. Materials and Methods

### 2.1. Patients

From January 2002 to December 2006 at our institute, 146 consecutive patients underwent hepatectomy for primary HCC, of which 97 patients were positive for HCV. Among these 146 and 97 patients, 96 of the total patients and 64 HCV-positive patients gave informed consent and their DNA was available for genotyping. Tumor stage and differentiation, or liver fibrosis stage, were diagnosed by pathological specialists according to the TNM stage definition proposed by the Liver Cancer Study Group of Japan [10], which agrees with the TNM classification system of the International Hepato-Pancreato-Biliary Association [11], and the Metavir score [12]. Curative resection was defined as negative for resection stump and for other organ metastasis. Patients were followed up monthly for the first 6 months, including assays of their peripheral blood for tumor markers such as AFP and ultrasonography, and enhanced CT every 6 months. The median follow-up time was 30.5 months. The current study was approved by the Ethics Committee of Kyushu University. 

### 2.2. DNA Extraction and Genotyping

Genomic DNA was extracted from the patients' nontumor liver tissues obtained at hepatectomy. The *MICA* genetic polymorphism (rs2596542) and the *DEPDC5* genetic polymorphism (rs1012068) were genotyped using the StepOnePlus real-time PCR system (Applied Biosystems). 

### 2.3. Statistical Analysis

All data were analyzed using JMP statistical software (SAS Institute, Cary, NC, USA). A chi-square test was performed for qualitative variables, and a Wilcoxon test was performed for quantitative variables. A Logrank test was performed for survival rates using the Kaplan-Meier method.

## 3. Results

### 3.1. *MICA* Genotypes and Their Clinical Association

Among the 64 HCV-positive patients, the *MICA* GG allele was seen in 30 and GA and AA were seen in 22 and 12 patients, respectively (minor allele frequency: 36.5%, which was comparable to 33.1–39.8% of those in literature [6]). The patients' backgrounds among these genotypes are outlined in Table 1. The preoperative, surgical, and tumor factors were similar among them. 

**Figure 1:**
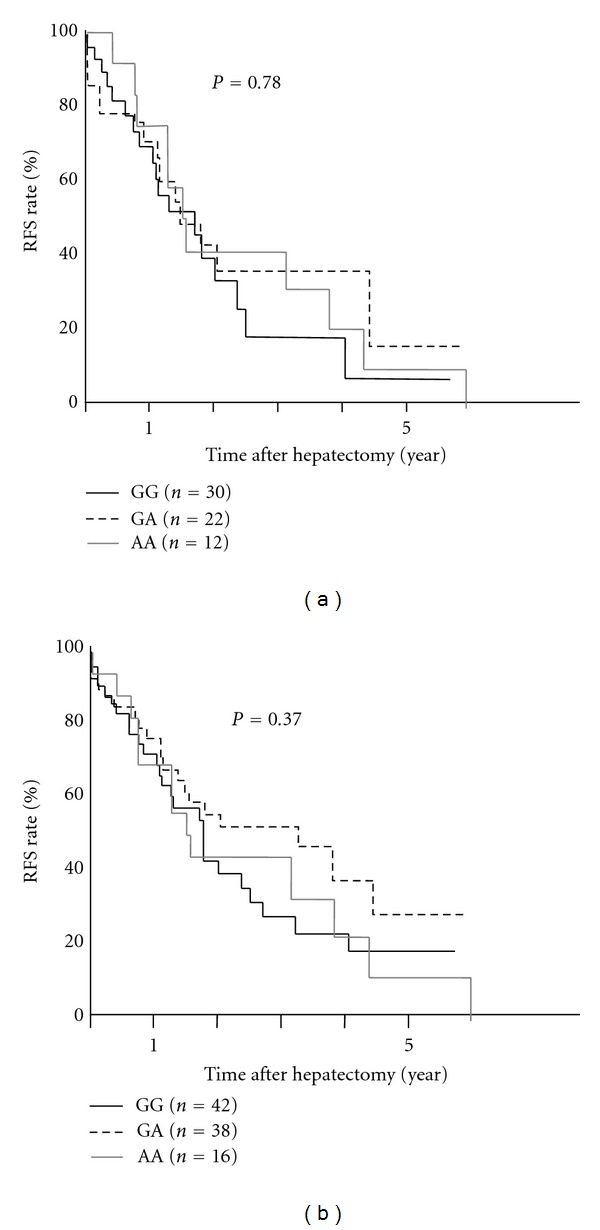
*MICA* genetic polymorphisms and HCC recurrence after hepatectomy. (a) The 5-year RFS rate among HCV-positive patients was not different between* MICA* GG (black solid line), GA (dotted line), and AA allele carriers (grey line). (b) The 5-year RFS rate among all patients was not different between each *MICA* genotype.

There was no difference in the 5-year recurrence-free survival rate (RFS) among HCV-positive patients among those carrying GG (20.7%), GA (31.8%), and AA (20.8%) alleles, respectively (*P* = 0.78, Figure 1(a)). No difference was seen among those who carried each genotype in all the patients following hepatectomy (22.8% in GG, 37.7% in GA, and 21.9% in AA carriers, *P* = 0.37, Figure 1(b)). 

### 3.2. *DEPDC5* Genotypes and Their Clinical Association

**Figure 2:**
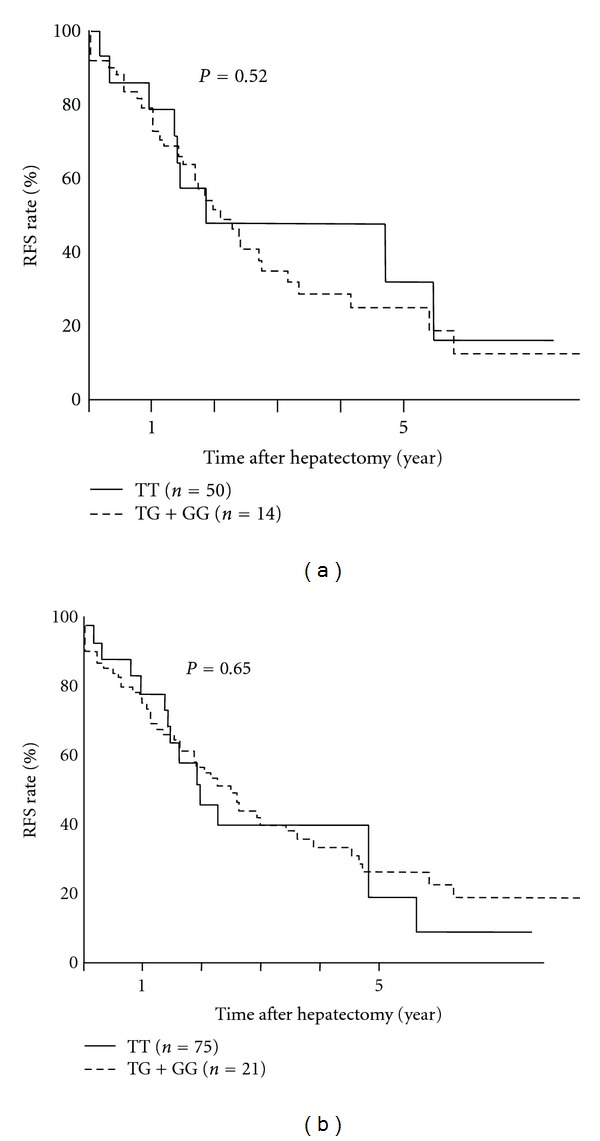
*DEPDC5* genetic polymorphisms and HCC recurrence after hepatectomy. (a) The 5-year RFS rate among HCV-positive patients was not different between *DEPDC5 *TT (solid line) and TG/GG (dotted line) allele carriers (grey line). (b) The 5-year RFS rate among all patients was not different between each *DEPDC5* genotype.

All 96 patients were then genotyped for *DEPDC5*. Only one patient carried the minor homozygote allele at the *DEPDC5* locus; therefore, the outcomes were compared between two genotypes (TT versus TG+GG, minor allele frequency was 11.7% which was similar to 12.1% of those in literature [7]). The HCV-positive patients' backgrounds between *DEPDC5* genotypes are summarized in Table 2. Similar to *MICA*, the preoperative, surgical, and tumor factors, except for liver fibrosis stages, were similar between each *DEPDC5* genotype. Among patients carrying the TG/GG allele, low fibrosis stages (F0-2) were more often observed than progressive stages (F3-4), compared with TT allele carriers (*P* < 0.05). There was no difference in RFS between each *DEPDC5* genotype among HCV-positive patients (23.8% versus 31.8%, *P* = 0.52, Figure 2(a)) or among all patients after hepatectomy (31.9% versus 22.4%, *P* = 0.95, Figure 2(b)). 

### 3.3. Clinical Features Associated with HCC Recurrence Following Hepatectomy

**Figure 3:**
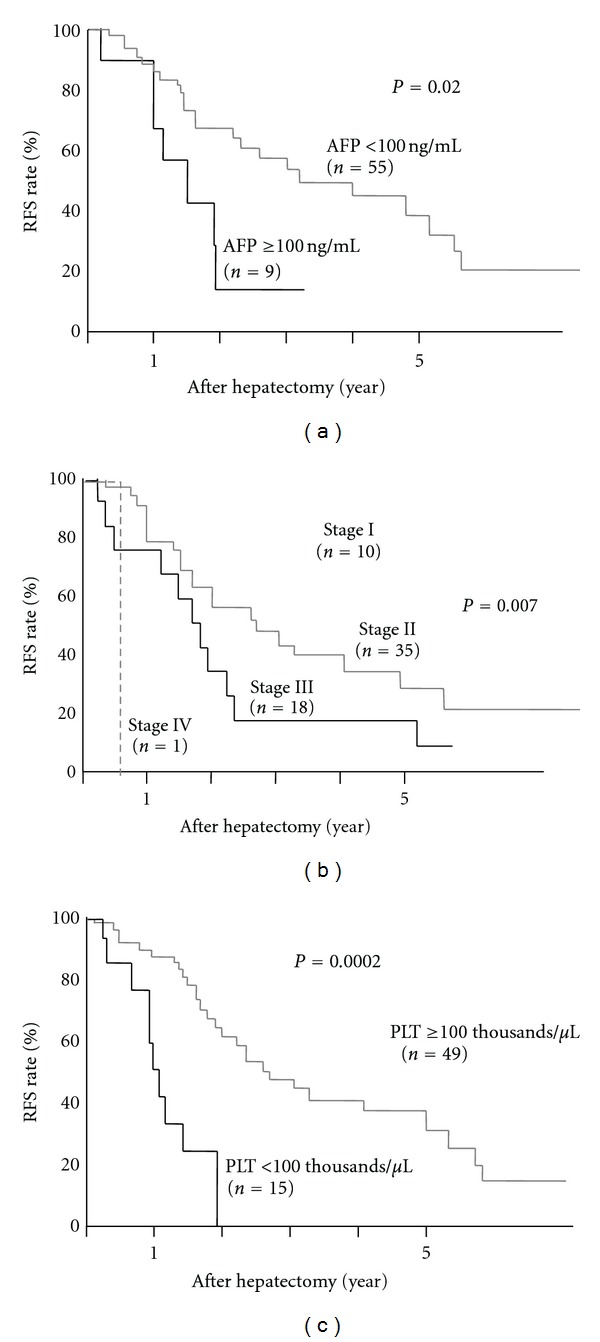
Clinical factors in HCV-positive patients and HCC recurrence after hepatectomy; (a) RFS rate was compared between two groups divided by AFP level of 100 ng/mL. (b) RFS was compared among pathological stages. (C) RFS was compared between those whose platelet count ≥100 thousands and <100 thousands.

To exclude the possibility that small number of cases led to no correlation, RFS rate was compared with alpha-fetoprotein (AFP) level, a well-known predictive factor for HCC recurrence [13]. As shown in Figure 3(a), RFS rate of patients whose AFP levels were equal to and over 100 ng/mL (*n* = 9) was significantly lower than those whose AFP levels were less than 100 ng/mL (*n* = 55) (5-year-RFS rate of 37.7% versus 13.9%, *P* < 0.05), even in a small number of cases. Similarly, the other clinical factors such as tumor stage and liver function (platelet count of over 100 thousands or not) also reflected significantly HCC recurrence after hepatectomy (Figures 3(b) and 3(c)). RFS rate of those whose tumor stage 1, 2, 3, and 4a was 68.2%, 34.6%, 16.8%, and 0%, respectively (*P* = 0.007). All the patients whose platelet count <100 thousands among HCV-positive showed HCC recurrence within five years, whereas 5-year RFS rate of those whose platelet count ≥100 thousands was 32.5% (*P* = 0.0002). Among these clinical factors, tumor stage, and platelet count were still significant factors associated with HCC recurrence after hepatectomy by multivariate analysis (Table 3).

## 4. Discussion

HCC recurrence among HCV-positive patients is a serious problem. Unlike other malignant tumors, multicentric recurrence can occur among these patients, even after curative resection of the liver. Identification of associated risk factors would permit early diagnosis and an opportunity for tailored therapy. We previously reported the significant association of hepatitis status or functions of the remnant liver with HCC recurrence, especially that caused by multicentric recurrence [4, 5]. Notably, tumor factors, including tumor size, histological grade, and alpha-fetoprotein levels, have been reported to not be significant risk factors for multicentric recurrence. Okamoto et al. [14] used a cDNA microarray to identify genes whose expression was significantly associated with multicentric HCC recurrence in HCV-positive liver parenchyma. The identification of noncancerous factors for HCC recurrence is urgently required. 

In this study, two SNPs in the *MICA* and *DEPDC5* genes, both of which had been first reported as genetic factors using genome-wide association study (GWAS) associated with HCV-induced HCC occurrence, were genotyped among HCC patients who had undergone hepatectomy. No correlation between these SNPs and HCC recurrence was detected. 

Although the molecular mechanism by which these two SNPs correlate with HCC progression remains unclear, *MICA* SNPs were suggested to affect antitumor immunity [8] and *DEPDC5* SNPs were reported to associate with other cancers, with or without HCV [9]. However, all patients, including HCV-negative ones, were also investigated, and no correlation was observed between the SNPs and recurrence. 

The problem that we are concerned with is that this study was limited to small sample size. The odds ratios of these SNPs with regard to susceptibility to HCC are as low as 1.39 for *MICA* and 1.75 for *DEPDC5*, as described in the previous reports [6, 7]. An investigation using a larger number of patients might lead to clarification of the association of these SNPs with HCC recurrence. The traditional factors for tumor recurrence such as AFP, however, still showed significant difference in RFS even among small number of patients enrolled in the current study. Clinical factors such as tumor marker, liver function, and such would rather be more beneficial to predict HCC recurrence so far, compared to such expensive and ethical problematic genotyping experience. 

Interestingly, the *DEPDC5* minor genotype was more susceptible to HCC development, even among those patients with low fibrosis stage (Table 2). In the current study, all the objective patients had HCC, which is different from previous reports that included non-HCC carriers and healthy subjects [6, 7]. This finding suggested that the *DEPDC5* genetic polymorphism at least affects susceptibility to HCC development in livers without pathological progression. In livers progressing to fibrosis or HCC, however, HCC recurrence may not depend on these genetic factors. 

## 5. Conclusions

In conclusion, neither *MICA* nor *DEPDC5* genetic polymorphisms might be a risk factor for HCC recurrence following hepatectomy. Patients carrying the *DEPDC5* minor allele were more susceptible to HCC development, even those with low fibrosis stages. 

## Figures and Tables

**Table 1:** Data among HCV-positive patients who underwent hepatectomy for HCC carrying GG, GA, and AA allele at rs2596542 (*MICA*).

rs2596542	GG (*n* = 30)	GA (*n* = 22)	AA (*n* = 12)	*P* value
Age (y), mean ± SD	68 ± 1	69 ± 1	72 ± 2	n.s
Sex (male/female), *n*	21/9	18/4	11/1	n.s
Platelet count (×10^4^/*μ*L), mean ± SD	13.1 ± 1.0	14.8 ± 1.2	13.8 ± 1.6	n.s
Total bilirubin level (mg/dL), mean ± SD	0.9 ± 0.07	0.9 ± 0.08	0.9 ± 0.1	n.s
Prothrombin time (%), mean ± SD	0/11/10	1/8/13	0/1/7	n.s
ICGR15 (%), mean ± SD	15.9 ± 1.4	16.5 ± 1.7	14.7 ± 2.3	n.s
Operative time (min), mean ± SD	369 ± 19	342 ± 23	322 ± 31	n.s
Intraoperative bleeding (mL), mean ± SD	779 ± 100	586 ± 118	617 ± 160	n.s
Maximum tumor size (cm), mean ± SD	3.7 ± 0.4	3.3 ± 0.5	3.3 ± 0.8	n.s
AFP level (ng/mL), mean (minimum–maximum)	797 (2.3–17156)	284 (2–4746)	907 (2.2–10368)	n.s
DCP level (mAU/mL), mean (minimum–maximum)	2484 (9–46561)	2710 (10–31134)	538 (9–3817)	n.s
Stage (I/II/III/IV), *n*	7/13/9/1	3/11/8/0	1/11/0/0	n.s
Tumor differentiation (well/moderate/poorly), *n*	5/22/3	7/13/2	3/7/2	n.s
Fibrosis stage (F0-2/F3-4), *n*	13/17	11/11	4/8	n.s

AFP: alpha-fetoprotein, DCP: des-gamma-carboxy prothrombin, HCC: hepatocellular carcinoma, ICGR15: indocyanine green retention at 15 minutes, and n.s: not significant.

**Table 2:** Data among HCV-positive patients who underwent hepatectomy for HCC carrying GG, GA, and AA allele at rs1012068 (*DEPDC5*).

rs1012068	TT (*n* = 50)	TG/GG (*n* = 14)	*P* value
Age (y), mean ± SD	69 ±1	71 ± 2	n.s
Sex (male/female), *n*	38/12	12/2	n.s
Platelet count (×10^4^/*μ*L), mean ± SD	13.5 ± 0.8	15.1 ± 1.5	n.s
Total bilirubin level (mg/dL), mean ± SD	0.9 ± 0.07	0.9 ± 0.1	n.s
Prothrombin time (%), mean ± SD	0/11/10	0/1/7	n.s
ICGR15 (%), mean ± SD	16.5 ± 1.4	15.7 ± 2.2	n.s
Operative time (min), mean ± SD	344 ± 15	357 ± 29	n.s
Intraoperative bleeding (mL), mean ± SD	631 ± 75	817 ± 146	n.s
Maximum tumor size (cm), mean ± SD	3.4 ± 0.5	4.0 ± 0.6	n.s
AFP level (ng/mL), mean (minimum–maximum)	2004 (2.0–5846)	250 (5.5–17156)	n.s
DCP level (mAU/mL), mean (minimum–maximum)	2429 (10–46561)	1348 (9–15526)	n.s
Stage (I/II/III/IV), *n*	7/28/14/1	3/8/3/0	n.s
Tumor differentiation (well/moderate /poorly), *n*	11/35/4	3/8/3	n.s
Fibrosis stage (F0-2/F3-4), *n*	19/31	8/6	<0.05

AFP: alpha-fetoprotein, DCP: des-gamma-carboxy prothrombin, HCC: hepatocellular carcinoma, ICGR15: indocyanin green retention at 15 minutes, and n.s: not significant.

**Table 3:** Multivariate analysis of factors affecting HCC recurrence after hepatectomy.

Variables	HR	95% CI	*P* value
AFP (≥100)	1.23	0.43–2.99	0.67
Stage1–4a	2.51	1.44–4.41	0.0012
Platelet count (<100 thousands/*μ*L)	5.85	0.07–0.43	0.0003

AFP: alphafeto protein, HCC: hepatocellular carcinoma, HR: hazard ratio, and CI: confidence interval.
